# Clinical Remarks on Surgical Cases Occurring in the Buffalo Hospital of the Sisters of Charity

**Published:** 1871-04

**Authors:** J. F. Miner


ART. II.—*Clinical Remarks on Surgical Cases occurring in the Buffalo Hospital of the Sisters of Charity.* By Prof. J. F. MINER, M. D.

REPORTED BY W. W. MINER, MEMBER OF THE CLASS.

CASE XV.—*Transplantation.*—A second case illustrating this new surgical procedure is here shown. The patient is a man of more than forty years of age, and has had an open syphilitic ulcer on his leg since his twenty-first year. The case is altogether one which belongs to our excellent hospital *interne*, M. J. Talbot, who commenced testing the value of the operation of skin grafting, immediately after the first operation of this kind was made in your presence. The success of the present case seems to be unexception-able, as all the transplanted germs have maintained their position and vitality, whereas generally, and as in our former case, some one or more of the transplanted germs fail to form living attachment to the part upon which they are implanted. It surely seems quite an accomplishment, that of causing the closure of a syphilitic ulcer which was of twenty years standing, and whose borders were formed of skin of such low vitality that it gave no promise of extending itself to any extent whatever.

The young boy whose heel was implanted with skin germs, after the denuded surface was entirely covered with skin, rose and walk-ed about the wards on crutches for two or three days. At the end of this time he was obliged to return to his bed because portions of the cicatricial covering of the heel came away, exposing a raw, bleeding surface. It was, however, noticed that the skin which sloughed away was altogether that formed by the natural process of cicatrization, while that which was produced by the operation of transplantation was not at all affected. The raw places were soon furnished with skin germs, and the boy is now in bed waiting for these germs to extend sufficiently to form the required covering. It is remarkable that such a difference exists in favor of the skin which is developed as the product of transplantation over that which was formed by the natural process of cicatrization. It seems that there is practically a limit to the extent to which skin will extend itself upon a raw surface, and the newly formed skin degenerates in character as it is removed from its center by proliferation. The sensation of touch is felt with normal acuteness in the skin of transplantation. This skin is probably not furnished with sweat or sebaceous secretion, or with hair; but it is of the natural thickness and affords proper protection to the parts which are covered by it.

CASE XVI.—*Exostosis of Right Os Innominatum.*—This gentleman, who is fifty-two years of age, presents an interesting growth on the front and right side of his pelvis, which extends also downwards in front of the femur. The firm, unyielding, bony growth, is of triangular form; its base measures six inches in breadth, and is attached to the anterior surface of the ilium, between its crest and the acetabulum. It is also about five inches in length, and projects downwards exactly in front and in a line with the femur, covering about three inches of the upper part of that bone. By pressing with one hand upon the great trochanter, and the outer surface of the femur, while motion of the thigh is made, it is determined with certainty that the movements of the femur and of the hip joint are altogether natural, and that the bony growth which projects downwards, forming a sort of triangular apron over this joint and the femur, is entirely distinct from these parts. The growth of this exostosis has taken place within two years, and at present its further continuance seems to be at an end. It has probably attained as large a size as it will reach, and no farther development will take place. This, I believe, is the manner of development usually manifested in exostosis.

These exostóses, or bony tumors, are liable to occur in any part of the bony structure of the body; they are sometimes of very large size; the scapula has been known to attain the size of a bushel measure; and I have in the museum of the college a bony tumor of the tibia, which is nearly one foot in diameter. The patient suffers from pain in the knee, which is caused by pressure upon the nerve trunks as they pass down in front of the femur and beneath this bony malformation. He is obliged to allay the pain by the use of morphine, frequently in good sized doses. His general health is very good indeed; his hair is quite grey, which is, he says, a family peculiarity. I see no reason for believing that this bony growth can be malignant in character. He has employed all sorts of things for the alleviation of his condition. He has tried preparations of iron, iodide of potassium, electro-magnetism and spiritualism: probably these latter are of an equal value with the former in the treatment of his case. In cases in which exsection of these bony enlargements is demanded, and can well be made, that operation may be instituted with success, or amputation of the affected part may rid one of the whole cause of trouble. In the present case, operative interference will not be advised until it is plainly necessitated.

